# Plant Volatile Organic Compounds Attractive to *Monolepta signata* (Olivier)

**DOI:** 10.3390/insects16121233

**Published:** 2025-12-06

**Authors:** Lun Li, Jiyu Cao, Zhiping Cai, Jing Chen

**Affiliations:** 1 Key Laboratory of Oasis Agricultural Pest Management and Plant Protection Resources Utilization, College of Agriculture, Shihezi University, Shihezi 832000, China; lilun096@163.com (L.L.); caoisgoodperson@outlook.com (J.C.); 2 Institute of Plant Protection, Xinjiang Uygur Autonomous Region Academy of Agricultural Sciences/Key Laboratory of Integrated Pest Management on Crops in Northwestern Oasis, Ministry of Agriculture and Rural Affairs/Xinjiang Key Laboratory of Agricultural Biosafety, Urumqi 830091, China

**Keywords:** *Monolepta signata*, plant volatiles, electrophysiology, olfactory behavior, field trapping, integrated pest management

## Abstract

Plant volatiles play a critical role in mediating insect host location behavior and offer eco-friendly alternatives for chemical control. *Monolepta signata* is a widespread phytophagous pest in East Asia that poses a significant threat to various crops. In this study, we evaluated the electrophysiological and behavioral responses of adult *M. signata* to 26 binary and ternary mixtures of 13 plant-derived volatile compounds extracted from cotton and corn leaves. Electroantennogram (EAG) recordings revealed that females exhibited the strongest responses to mixtures 20, 23, and 12, whereas males were the most responsive to mixtures 26, 19, and 4. Y-tube olfactometer assays showed significant behavioral attraction of females to mixtures 1, 20, 23, and 26, and males to mixtures 19, 22, and 23. Field trials demonstrated that mixtures 22, 23, and 26 attracted significantly more adults than the control, with mixture 23 (*α*-phellandrene + *trans*-2-hexen-1-ol + 1-heptene) achieving the highest trap capture. These findings highlight the potential of specific volatile blends, especially ternary mixtures, as effective attractants of *M. signata*, and support their application in environmentally sustainable pest monitoring and management strategies.

## Simple Summary

*Monolepta signata* (Olivier) is a beetle that damages important crops in East Asia, such as cotton and corn. Traditional control methods rely heavily on chemical pesticides, which can harm the environment and cause pest resistance. In this study, we explored whether plant volatiles, natural smells released by plants, could be used to attract beetles. We created 26 different scent mixtures using common plant odors and tested how the beetles reacted to them in the laboratory and the field. Some mixtures were very attractive to the beetles, particularly those containing three different odors. One mixture, made from *α*-phellandrene, *trans*-2-hexen-1-ol, and 1-heptene, attracted the most beetles in field traps. Our findings suggest that plant-based scent mixtures can be developed as safer and more sustainable ways to monitor or control this pest, without relying on harmful pesticides.

## 1. Introduction

Plants release a wide variety of volatile organic compounds (VOCs) from their leaves, flowers, and fruits as part of their interactions with the environment [1]. Plant VOCs are low-molecular-weight, highly volatile chemicals released into the atmosphere, which disperse rapidly and serve as key cues for herbivorous insects [2,3]. More than 1700 volatile compounds have been identified in over 90 plant families, including hydrocarbons, alcohols, aldehydes, ketones, esters, organic acids, and terpenes. This represents a wide diversity of plant-derived secondary metabolites [4]. Numerous studies have demonstrated that plant volatiles can significantly influence insect behavior, such as host location, mating, and oviposition [5,6,7,8]. Several studies have shown that host-derived volatiles can effectively attract phytophagous pests, and can be applied to the management of agricultural and forestry pests such as *Ectropis obliqua*, *Empoasca onukii*, and *Adelphocoris* species [9,10,11].

*Monolepta signata* (Olivier, 1808) (Coleoptera: Chrysomelidae) is a herbivorous agricultural pest widely distributed in China [12,13]. This insect feeds directly on the leaves of the host plants, and its host range includes various plant families such as Gramineae, Malvaceae, Leguminosae, Cruciferae, Rosaceae, Ulmaceae, and Salicaceae. It mainly affects cotton, corn, rice, sorghum, and potatoes, leading to reduced crop yields [14]. It was first reported in the Xinjiang Uygur Autonomous Region in the late 1990s and has since caused significant damage and economic losses to major crops such as cotton and corn, becoming one of the most important agricultural pests in the region [15,16,17]. Currently, chemical treatment is the primary strategy for controlling *M. signata*; however, this has led to problems such as resistance, food safety concerns, and environmental pollution [18]. Therefore, there is an urgent need to reduce the use of pesticides, develop environmentally friendly measures to control adult pests in fields, and protect crops from *M. signata* infection.

An effective semiochemical lure for population monitoring is important in integrated pest management (IPM) [19]. Consequently, the identification of bioactive compounds from host plants is crucial for understanding host selection behavior and olfactory function in phytophagous insects, and it is crucial to develop effective host plant volatile attractants [20]. Insects rely on highly sensitive olfactory systems to detect plant volatiles and use these systems to locate suitable hosts. Previous studies have shown that the antennae of *M. signata* contain 114 olfactory-related genes and seven types of sensilla, three of which exhibit porous structures associated with semiochemical perception [21,22]. These volatiles are typically released as complex blends, and most insects respond to a mixture rather than single compounds for accurate host recognition [6,23,24,25,26]. *Aulacophora foveicollis* females respond positively to volatile blends of *Solena amplexicaulis* fruit because of the synergistic action of nonanal and *(E, Z)*-2, 6-nonadienals [27]. Vine weevil adults are attracted to a mixture of seven plant volatiles, although either no responses or negative responses were recorded for each of these compounds when presented individually [28]. For volatiles in the seeds of four legume varieties, the attraction effect on the important leguminous insect pest *Callosobruchus maculatus* was significantly enhanced when these compounds were formulated in various proportion [29]. *Cis*-3-hexen-1-ol from tobacco plants induces oriented flight in *Phthorimaea operculella*, whereas nonanal, decanal, decane, methyl hexadecanoate, and related volatiles stimulate oviposition in female adults [30]. Therefore, it is important to study the effects of mixed components in host plants on insect behavior [20].

Previous studies have demonstrated that under laboratory conditions, both the type and concentration of individual VOCs can influence the behavior of *M. signata*. Among the various plant volatiles, peppermint essential oil exhibited the strongest repellent effect, with a repellency rate of 74%, whereas geraniol showed the strongest attractive effect, with a luring rate of 70% [31]. In a study involving seven cotton and corn derived volatiles, *M. signata* females were attracted to *β*-ionone at a concentration of 10 μg/mL; however, they were repelled by *γ*-terpinene and caryophyllene oxide. In contrast, males were attracted by *γ*-terpinene and D-limonene; however, they were repelled by *β*-ionone [32]. In our previous studies, Dragosantol and *α*-pinene from cotton and corn leaves were identified to have significant attraction to *M. signata* adults [33]. Currently, we have detected 1-heptene, aromadendrene, *trans*-2-hexen-1-ol, *α*-phellandrene, *α*-caryophyllene, and hexadecene from cotton leaves, *α*-farnesene and heptadecane from corn leaves, and common *β*-ocimene, *trans*-2-hexenal-al, *(Z)*-3-hexen-1-ol (leaf alcohol), *β*-pinene, nerolidol, and 3-methylpentanal from cotton and corn leaves using GC-MS (unpublished data). Several studies have shown that a mix of host volatile species in the field is more effective in attracting insects than a single host volatile [34,35,36]. However, few studies have investigated the behavioral responses of mixed components in host plants to *M. signata*, and there is limited research on the use of plant volatiles to attract *M. signata* for large-scale biological control in the field.

Although several plant volatiles have been found to elicit electrophysiological or behavioral responses in *M. signata* under laboratory conditions, little is known about the effects of volatile blends on this species. To fill this knowledge gap, this study aimed to (1) evaluate the electrophysiological and behavioral responses of *M. signata* adults to 26 mixtures of plant volatiles using electroantennogram (EAG) recordings and Y-tube olfactometer assays, and (2) identify the most attractive blends for potential application in field trapping systems.

## 2. Materials and Methods

### 2.1. Insects

*M. signata* adults were collected from cotton fields in Shihezi City, Xinjiang Uygur Autonomous Region, China (44°32′85″ N; 85°98′33″ E). The fields are sparsely surrounded by weeds. The collected adults were reared on fresh cotton leaves in growth chambers set at 25 ± 1 °C, 50–70% relative humidity, 2000 lx light intensity, and a 16:8 h light: dark photoperiod.

### 2.2. Chemicals

The standard chemical samples used in this study are listed in Table 1.

### 2.3. Preparation of Plant Volatile Mixtures

The nine VOCs used in this study were selected from the 14 volatiles previously identified in cotton leaves (*Gossypium hirsutum*) and corn leaves (*Zea mays*) by headspace collection and GC–MS analysis (unpublished data). Compounds were selected based on their consistent detection in both host plants and their reported electrophysiological or behavioral activities in Coleopteran species. In the preliminary experiments, all 14 compounds were tested in EAG dose–response assays with *M. signata*, and the nine compounds that elicited the strongest responses at their optimal concentrations selected for subsequent experiments (Figure S1).

Nine VOCs, namely, alcohols, aldehydes, terpenoids, and hydrocarbons, were classified according to their major functional categories. Following previously reported attractant formulation strategies [25], mixtures were designed by combining compounds with different functional groups (e.g., alcohol + aldehyde, terpene + aldehyde + hydrocarbon) to explore potential additives or synergistic effects. To ensure that each compound in a mixture reached its individually determined optimal EAG concentration, each individual compound was first diluted in liquid paraffin to an appropriate stock concentration. Binary and ternary mixtures were then prepared by combining equal volumes of the appropriately concentrated stocks so that the final concentration of each compound in the mixture equaled its optimal EAG concentration (10 μL/mL or 1 μL/mL). All mixtures were freshly prepared immediately prior to behavioral and electrophysiological assays (Table 2).

### 2.4. Determination of EAG Responses of M. signata to Mixtures of Plant Volatiles

The EAG responses of *M. signata* were recorded using an EAG detection system following the method described by Liu [31]. The system of EAG, including IDAC-2, CS-55, EAG probe (Syntech, Hilversum, The Netherlands), Faraday cage, and recording software, was named GC-EAD (version 1.2.5). Before each test, the sex of the adult *M. signata* was identified and the individuals were starved for at least 4 h. The airflow system was then checked to ensure normal operation, and it was confirmed that the distilled water in the humidifying bottle did not enter the airflow lines. For each compound or mixture, 20 μL was applied in a “Z” shape onto a filter paper strip (length 50 mm, width 5 mm), which was then inserted into a Pasteur pipette. Each Paster tube was used only once to test each compound. A humidified airflow of 500 mL/min was directed through a pipette and positioned 1 cm from the antenna. The stimulus was delivered at 100 mL/min for 1 s, with an interval of at least 60 s between stimulations to prevent adaptation. N-hexane served as the reference, and liquid paraffin was used as the control. The test sequence was as follows: liquid paraffin, n-hexane, test volatile mixture, n-hexane and liquid paraffin. Each mixture was tested in numerical order, and the responses were recorded from six different antennae per treatment. To minimize variation, only active and undamaged adult *M. signata* were selected for testing. Each volatile mixture was tested using six biological replicates, with one antenna per beetle. None of the antennas were used more than once. All tests were conducted between 25 ± 1 °C from 10:00 to 20:00.

### 2.5. Bioassay of Behavioral Response of M. signata to Mixtures of Plant Volatiles

The behavioral responses of *M. signata* adults to plant volatile mixtures were evaluated using a glass Y-tube olfactometer. The olfactometer consisted of a main glass tube (1.5 cm; length: 15 cm) with two arms (10 cm each) that diverged at an angle of 70°. Each arm was connected to a glass odor source chamber via Teflon tubing. The apparatus was illuminated by two 20 W fluorescent lamps (light intensity: 2000 lx) and maintained under controlled environmental conditions (25 ± 1 °C, 60 ± 5% RH). Constant airflow (200–300 mL/min, LZB-3WB, Changzhou Kede thermal instrument Co., Ltd., Changzhou, China) was provided by two atmospheric samplers (QC-1S, Scientific Institute of Labour Protection, Beijing, China) through a drying tower (250 mL) filled with activated charcoal. For each assay, 20 μL of a volatile mixture was applied to a filter paper strip and placed in one odor source bottle, while the other bottle contained a strip treated with an equal volume of liquid paraffin (control). All Y-tube bioassays were conducted in a dedicated insect behavioral laboratory under odor-controlled conditions. The experimenter remained outside the airflow path during the tests to prevent odor contamination.

To minimize variation, only active and undamaged adults of *M. signata* were selected for testing. Individual adults were starved for at least 4 h before the bioassay, then placed at the base of the Y-tube olfactometer for observation for a 10 min period. A choice was recorded when an insect entered a branch tube by more than two-thirds of its length and remained there for at least 30 s, otherwise, it was recorded as “no choice.” The odor-source positions were switched every five insects to prevent positional bias, and a new Y-tube was used every 10 insects. Eighty female and 80 male *M. signata* adults were tested in each treatment, and each was used only once. All assays were conducted at 25 ± 1 °C from 10:00 to 20:00.

### 2.6. Field Attraction

The field trial was conducted in an 8 hm^2^ cotton field in Shihezi, Xinjiang Uygur Autonomous Region, China, during the flowering stage when *M. signata* adults were predominant. The field population density was estimated from the control traps, with three traps (15 m apart) capturing an average of approximately three adults per trap over the 14-day survey period. New moth traps equipped with polyethylene sustained-release bottles (Beijing Zoje Sifang Biotechnology Co., Ltd., Beijing, China) were used. Each mixture (1 mL) was loaded into identical polyethylene slow-release bottles (wall thickness 0.8 mm, mouth diameter, 7 mm) to standardize evaporation conditions among treatments, while control traps contained the same volume of liquid paraffin. The traps were suspended slightly above the cotton canopy, with a minimum distance of 15 m between adjacent traps. A randomized complete block design was used, with three independent blocks established within a single 8 hm^2^ cotton field. Each block contained all treatments, and the blocks were separated by more than 50 m to ensure independent replicates and to minimize cross-interference among the volatile sources. The number and sex of *M. signata* adults captured were recorded every two days throughout the trapping period. Sex identification was performed under a stereomicroscope based on the abdominal morphology, and all captured individuals were removed after each count. No pesticides were used during the experimental period.

### 2.7. Data Analyses

The EAG response of *M. signata* to plant-derived volatile mixtures was calculated using the following formula:

A = (B − C)/(D − C) × 100%,

where A is the relative EAG response to a given stimulus; B is the raw EAG amplitude in response to the stimulus; C is the mean EAG response to liquid paraffin (recorded before and after the stimulus); and D is the mean EAG response to n-hexane (recorded before and after the stimulus) [37]. Differences in EAG responses among treatments were analyzed using one-way analysis of variance (ANOVA), followed by least significant difference (LSD) multiple range tests for post hoc comparisons. Differences between the male and female EAG responses in the same mixture were analyzed using an independent samples *t*-test.

For behavioral assays, the null hypothesis assumed no preference by *M. signata* adults for either arm of the olfactometer (i.e., a 1:1 distribution of responses). This was tested using chi-square (*χ*^2^) goodness-of-fit tests, excluding non-responding individuals. Bonferroni adjusted *p*-values are also provided as references. Both raw and adjusted *p*-values are reported.

Trap capture data from the field experiments were also analyzed using one-way ANOVA to assess the effects of the different volatile mixtures. All statistical analyses were performed using SPSS software (version 20.0; SPSS Inc., Chicago, IL, USA).

## 3. Results

### 3.1. Electroantennogram Responses of M. signata to Plant Volatile Mixture

**Figure 1:**
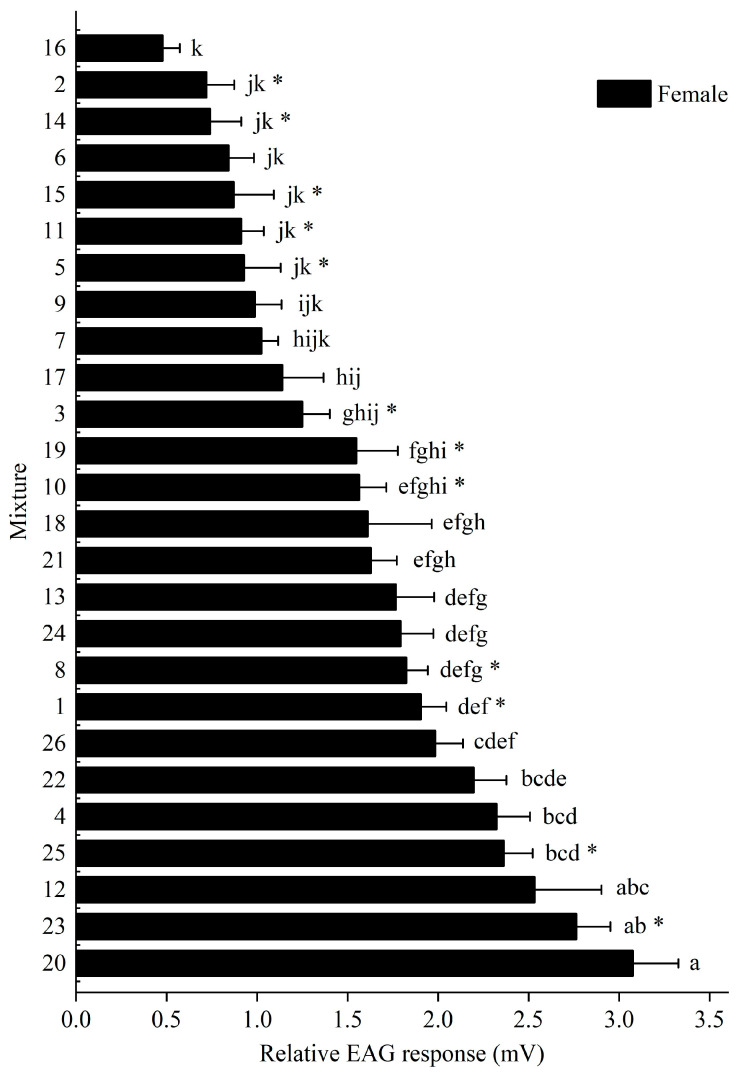
Electroantennogram (EAG) response of female *M. signata* to 26 mixtures of plant volatiles. These data represent the mean ± SE, while the by letters indicate the results of mixed volatiles on the different types by Duncan’s multiple range test. * indicates a significant difference (*p* < 0.05, independent-samples *t*-test) between female and male antennae in response to the same volatile compound.

The EAG amplitudes of female *M. signata* to the 26 plant-derived mixtures differed significantly among some treatments (Figure 1). Mixture 20 elicited the strongest response (3.076 ± 0.251 mV), followed by mixtures 23 (2.764 ± 0.188 mV) and 12 (2.534 ± 0.369 mV). Eight mixtures (1, 4, 12, 20, 22, 23, 25, and 26) elicited responses greater than 1.905 mV and were therefore selected as candidate attractants for subsequent behavioral assays (Figure 1).

**Figure 2:**
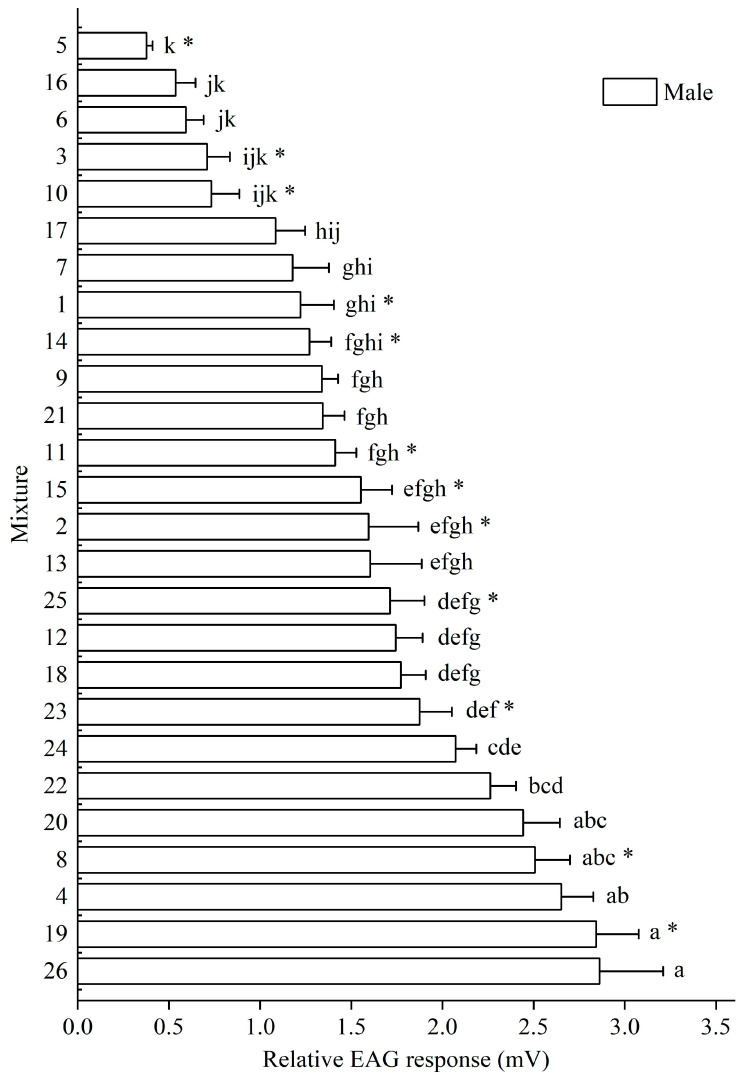
Electroantennogram (EAG) response of male *M. signata* to 26 plant volatile mixtures. These data represent the mean ± SE, while the letters indicate the results of mixed volatile on the different types by Duncan’s multiple range test. * indicates a significant difference (*p* < 0.05, independent-samples *t*-test) between female and male antennae in response to the same volatile compound.

Similarly, males showed the highest response to mixture 26 (2.861 ± 0.449 mV), followed by mixtures 19 (2.843 ± 0.233 mV) and 4 (2.652 ± 0.175 mV). Eight mixtures (4, 8, 19, 20, 22, 23, 24, and 26) elicited responses greater than 1.874 mV, and were chosen as candidate attractants for behavioral evaluation. Notably, mixtures 4, 20, 22, 23, and 26 elicited consistently strong responses in both sexes and were, therefore, considered key candidates for further analysis (Figure 2).

Both male and female antennae responded to all tested mixtures, with varying response amplitudes. There were no significant differences in the EAG responses between males and females for most mixtures (*p* > 0.05), except for mixtures 1 (*F* = 2.547, df = 10, *p* = 0.014), 2 (*F* = 2.477, df = 10, *p* = 0.019), 3 (*F* = 0.017, df = 10, *p* = 0.021), 5 (*F* = 5.711, df = 10, *p* = 0.023), 8 (*F* = 3.346, df = 10, *p* = 0.012), 10 (*F* = 0.162, df = 10, *p* = 0.003), 11 (*F* = 0.208, df = 10, *p* = 0.015), 14 (*F* = 0.130, df = 10, *p* = 0.029), 15 (*F* = 1.193, df = 10, *p* = 0.035), 19 (*F* = 0.001, df = 10, *p* = 0.003), 23 (*F* = 0.081, df = 10, *p* = 0.006), and 25 (*F* = 0.595, df = 10, *p* = 0.090), which showed significant sex-based differences (Figure 1 and Figure 2).

### 3.2. Behavioral Responses of M. signata to Mixtures of Plant Volatiles

**Figure 3:**
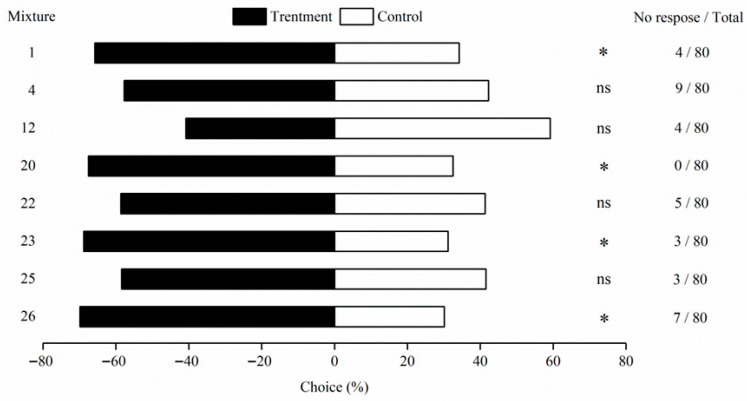
Behavior response of *M. signata* of female adults to eight plant volatile mixtures. The data obtained were all tested by *χ*^2^, * indicates significant difference (*p* < 0.05), and ns indicates no choice.

Eight plant-derived attractants screened using EAG were further evaluated in olfactory behavioral assays with *M. signata*. In females, formulations 1 (*χ*^2^ = 3.981, *p* = 0.046), 20 (*χ*^2^ = 5.055, *p* = 0.025), 23 (*χ*^2^ = 5.726, *p* = 0.016), and 26 (*χ*^2^ = 6.248, *p* = 0.012) elicited significant attraction (*p* < 0.05), with response rates of 65.79%, 67.50%, 68.83%, and 69.86%, respectively. Although formulation 1 did not remain significant after Bonferroni correction (adjusted *p* = 0.067), it still exhibited a consistent trend of attraction (Table 3). Among all formulations, formulation 26 showed the strongest response. In contrast, formulations 4 (*χ*^2^ = 0.908, *p* = 0.341), 12 (*χ*^2^ = 1.333, *p* = 0.248), 22 (*χ*^2^ = 1.171, *p* = 0.279), and 25 (*χ*^2^ = 1.126, *p* = 0.298) did not produce significant effects (*p* > 0.05) (Figure 3).

**Figure 4:**
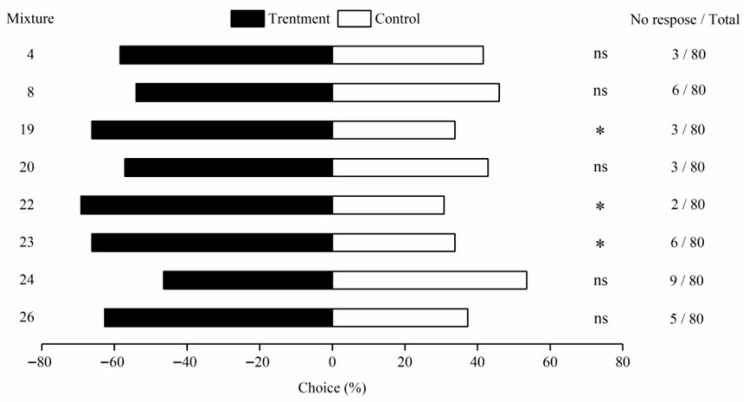
Behavior response of *M. signata* of male adults to eight mixtures of plant volatiles. The data obtained were all tested by *χ*^2^, * indicates significant difference (*p* < 0.05), and ns indicates no choice.

In males, significant attraction was observed for formulations 19 (*χ*^2^ = 4.244, *p* = 0.039), 22 (*χ*^2^ = 6.061, *p* = 0.014), and 23 (*χ*^2^ = 4.144, *p* = 0.042), with response rates of 66.23%, 69.23%, and 66.22%, respectively; formulation 22 showed the highest response. Although formulations 19 and 23 did not remain significant after Bonferroni correction (adjusted *p* = 0.058 and 0.061), they still exhibited a consistent trend of attraction (Table 3). No significant effects were detected for formulations 4 (*χ*^2^ = 1.126, *p* = 0.289), 8 (*χ*^2^ = 0.253, *p* = 0.615), 20 (*χ*^2^ = 0.805, *p* = 0.370), 24 (*χ*^2^ = 0.187, *p* = 0.666), and 26 (*χ*^2^ = 2.522, *p* = 0.112) (*p* > 0.05) (Table 3, Figure 4).

### 3.3. Evaluation of the Field Level Trapping

The trapping efficacy of the six plant-derived attractants against *M. signata* adults showed an initial increase followed by a decline. Mixtures 1, 19, 22, 23, and 26 peaked on 16 July, whereas formulation 20 peaked on 18 July. Mixtures 22 and 23 captured significantly more adults than the control on 16 July, while mixtures 20 and 26 captured more adults on 18 July (*p* < 0.05). Notably, only mixture 23 maintained significant attractiveness on 24 July (Table 4).

**Figure 5:**
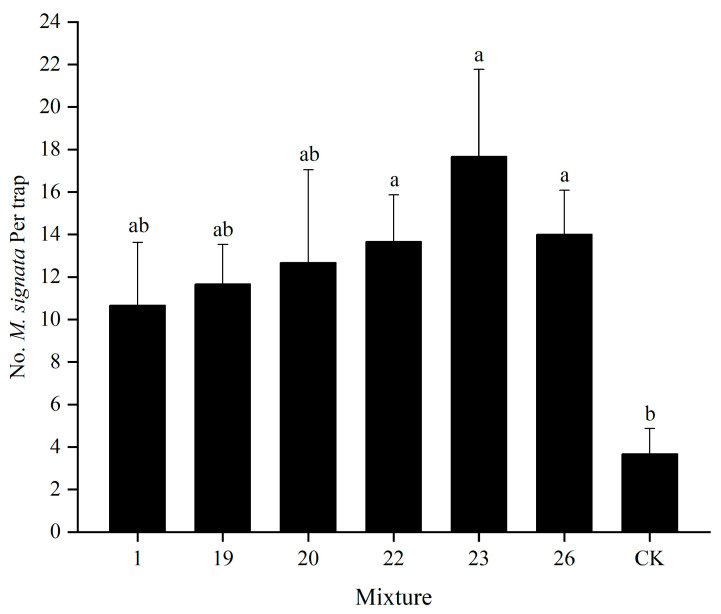
Field trapping effect of six different mixtures of plant volatiles on the *M. signata* adults. Data are presented as the means ± SEMs. Different lowercase indicates significant difference at the *p* < 0.05 level.

Field trials demonstrated that mixtures 22, 23, and 26 captured significantly more *M. signata* adults than the control (*p* < 0.05), with mixture 23 showing the highest capture (17.7 ± 4.1 adults per lure), followed by mixtures 26 (14.0 ± 2.1) and 22 (13.7 ± 2.2). In contrast, mixtures 1, 19, and 20 did not differ significantly from the control (*p* > 0.05; Figure 5).

Both female and male *M. signata* adults were captured by all the mixtures, however females were trapped in significantly higher numbers than males (Figure S2). Mixture 23 had the highest female catch and overall attractiveness, followed by mixtures 1 and 26. Mixture 1 attracted significantly more females than males (*p* < 0.05), whereas no significant sex-related differences were observed with the other five volatile plant mixtures.

## 4. Discussion

In this study, we identified specific plant-derived volatile mixtures that elicit strong antenna and behavioral responses in *M. signata*, and demonstrated that several of these blends, particularly ternary formulations, also exhibit practical attractiveness under field conditions. These findings indicate that *M. signata* may rely on complex host–plant volatile combinations rather than simple two-component combinations, underscoring the ecological importance of blend synergy in host–location behavior. Most insects detect complex volatile blends emitted by host plants via their antennae, and *M. signata* similarly possesses abundant olfactory receptors and antennal sensilla that enable the detection of such cues [21,22,38]. Although our use of six antennae per treatment follows common practice in EAG studies [37], future work could benefit from increased replication and from testing multiple concentrations of each volatile mixture to better assess dose-dependent effects. In behavioral assays, the mixture 26 (*trans*-2-hexen-1-al + 1-heptene + *trans*-2-hexen-1-ol) was most attractive to females, followed by mixture of 23 (*α*-phellandrene + *trans*-2-hexen-1-ol + 1-heptene), mixture of 20 (leaf alcohol + *trans*-2-hexen-1-ol + 1-heptene), and mixture of 1 (*β*-pinene + leaf alcohol). For males, the most attractive was mixture 22 (*β*-pinene + *trans*-2-hexen-1-al + leaf alcohol), followed by mixture 19 (*β*-pinene + *α*-phellandrene + *trans*-2-hexen-1-al), and mixture 23 (*α*-phellandrene + *trans*-2-hexen-1-ol + 1-heptene). These results indicate that specific mixtures can elicit sex-specific behavioral responses, highlighting the potential to optimize attractant formulations for targeted monitoring or management of *M. signata* in the field. Previously reported single-compound attractants such as geraniol achieved an attraction rate of 64 ± 8.94% at concentrations of 10^−1^ mol·L^−1^ [31], which is lower than the attraction rates obtained for the mixtures formulated in this study (mixtures 1, 19, 20, 22, 23, and 26). This difference may be related to the concentrations and release dynamics of the volatiles used [33]. Overall, ternary mixtures consistently outperformed binary combinations, supporting previous findings that multicomponent blends are more effective at attracting insects than single or dual compounds [34,35,36]. This may be because insects rely on the antennal detection of complex volatile profiles as environmental cues that guide their host-seeking behavior [28,37].

This study also revealed sex-specific differences in both the EAG and behavioral responses of *M. signata* adults to the same volatile mixtures. In the field trials, females were captured in higher numbers than males for all mixtures, except mixtures 19 and 22. Although the same volatiles were tested, males and females often exhibited different EAG responses [39], as reported for *Brontispa longissima* [40] and *Monochamus alternatus* [41]. A similar pattern was observed in *M. signata*, where sex-specific responses to identical volatile mixtures likely reflect divergent reproductive strategies and dietary requirements, particularly in host location and oviposition [42]. These findings further indicate that insect olfactory systems are sufficiently sophisticated to detect subtle variations in plant volatile profiles under natural conditions [38]. In addition, the behavioral responses of male and female *M. signata* suggested that modifying a single component within a volatile blend may influence its attractiveness, a phenomenon also noted in previous studies [43,44,45]. In our assays, *trans*-2-hexen-1-ol and 1-heptene appeared to be the key compounds driving female attraction, with leaf alcohol, *α*-phellandrene, *trans*-2-hexen-1-al, and *β*-pinene serving as secondary enhancers. In contrast, *β*-pinene and *trans*-2-hexen-1-al likely played primary roles in male attraction, while *α*-phellandrene and leaf alcohol may act as supporting contributors. Such primary compounds are often indispensable for insect attraction because their replacement typically abolishes the response, whereas altering the secondary components tends to diminish it, which is consistent with the observations of Thöming and Knudsen [46]. These findings highlight the importance of disentangling the roles of individual and combined volatiles, which is essential for identifying key attractants and developing environmentally friendly tools for pest monitoring and control [47]. However, as field-collected adults of unknown age and mating status were used in this study, these factors may have contributed to individual variations in antennal or behavioral responses [48]. Future studies using laboratory-reared insects under standardized physiological conditions will improve the comparability and reproducibility of the results.

In the field-trapping experiment, the attractiveness of all six mixtures declined over time, which is consistent with the typical release pattern of field attractants [30,49,50]. Among them, the mixture 23 (*α*-phellandrene + *trans*-2-hexen-1-ol + 1-heptene) tended to attract more insects on average. During the penultimate field survey, only mixture 23 showed a significantly higher trap catch than the control, suggesting that it may have maintained attractiveness longer than the other mixtures under the tested conditions. The release rates of the mixtures were not measured directly, which limited the precise interpretation of their relative effectiveness. Future work will aim to quantify the release profiles of each formulation to improve field evaluation accuracy. Our results showed that although some mixtures, such as mixture 20, elicited strong electroantennographic and behavioral responses in the laboratory, their field attractiveness was much weaker. This discrepancy suggests that EAG and behavioral responses alone may not fully reflect host selection behaviors under natural conditions [51,52]. The discrepancies observed between the laboratory and field results may have occurred due to differences in environmental and ecological conditions. In laboratory olfactometers, insects are exposed to pure, high-concentration odor streams without competing stimuli, whereas in the field, the presence of abundant background volatiles from natural hosts, fluctuating wind direction, temperature, and humidity can dilute or mask synthetic odors [9]. Moreover, odor release dynamics and blend ratios may differ between laboratory dispensers and field traps, leading to altered perceptions or behavioral responses [10]. Similar inconsistencies between laboratory and field performance have been widely reported in chemical ecology studies [48,53].

Environmental variables such as blend concentration, background odors, temperature, humidity, and surrounding vegetation further influence the attractiveness of volatiles in the field [53,54]. In the present field trials, these parameters remained relatively stable (26–32 °C, 50–70% RH, <1.5 m/s wind), yet even small fluctuations could affect volatile release rates and insect activity [50]. Therefore, although our results reflect typical summer conditions in the cotton fields, they should be interpreted with caution. The nine volatiles used in this study were originally identified in both cotton and corn leaves. However, since field validation was performed only in cotton, additional experiments in corn fields are needed to confirm whether the observed behavioral patterns are consistent across different host habitats. Future studies should focus on optimizing lure release rates, trap design, and placement, as well as refining release devices and formulations to maintain stable volatile emissions under field conditions [10,20]. To the best of our knowledge, no commercial attractants have been developed for *M. signata*, highlighting the practical potential of the plant volatile mixtures identified in this study. In addition, replicated field experiments across different climatic regions are essential to validate the consistency and applicability of attractant formulations in diverse environmental settings. Despite these challenges, plant-derived synthetic attractants remain promising tools for monitoring and managing leaf beetle populations as they can attract both sexes and contribute to integrated pest management programs [20].

Three types of attractant formulations were selected in this study based on the volatile compounds emitted from cotton and corn leaves. These compounds, including leaf alcohol, *β*-pinene, *trans*-2-hexen-1-al, *α*-phellandrene, *trans*-2-hexen-1-ol, and 1-heptene are common green leaf volatiles widely found in plants and play important roles in mediating interactions between host plants and phytophagous insects [55]. Leaf alcohol has been reported to enhance courtship behavior in certain Lepidoptera and Coleoptera species [56,57,58], and also shows moderate attractiveness to *Anoplophora glabripennis* [59]. *β*-pinene, as the main component in attractants, significantly attracts *Monochamus alternatus* [60]. Attractants containing *trans*-2-hexen-1-al strongly attract *Asias halodendri* [61,62], while *α*-phellandrene-based attractants are effective against *Ips pini* in the field [63]. *Trans*-2-hexen-1-ol attracts *Chlorophorus diadema* and *Curculio chinensis* [64,65], and 1-heptene elicits strong EAG responses in *A. glabripennis* [66]. The volatile components identified in this study are consistent with those reported previously and likely represent the key constituents responsible for attracting *M. signata*.

Although this study identified several plant-derived volatile mixtures with strong laboratory and field attractiveness (mixtures 22, 23, and 26), we acknowledge several limitations that should be addressed in future research. First, the actual release dynamics of volatile blends from polyethylene slow-release dispensers were not quantitatively monitored. Techniques such as dynamic headspace sampling and GC–MS analysis would allow characterization of the types, ratios, and release rates of volatiles under both laboratory and field conditions, helping verify whether the “odor profile” tested in the laboratory corresponds to that emitted in the field. Second, environmental factors such as crop growth stage, surrounding vegetation, and background odors may influence lure performance and partially explain the discrepancies between laboratory and field results. Incorporating these factors in future field trials will help improve the robustness and practical reliability of these attractant formulations. Overall, our findings provide a basis for the development and application of plant-based attractants for *M. signata* and may contribute to environmentally friendly pest management strategies, including biological control.

## 5. Conclusions

In this study, we identified and evaluated plant-derived volatile mixtures that elicited strong electrophysiological and behavioral responses in *Monolepta signata*. Among them, the mixture 23 (*α*-phellandrene + *trans*-2-hexen-1-ol + 1-heptene) was consistently attractive to *M. signata* and showed superior field trapping performance. Based on these findings, mixture 23 was granted a Chinese invention patent for its use as an attractant formulation for *M. signata* (Patent No. ZL202110177367.0). This formulation can be integrated into slow-release polyethylene lures and used in field-monitoring traps to facilitate the early detection and control of *M. signata* infestations. Future research should optimize dispenser design, assess long-term stability, and evaluate its compatibility with integrated pest management (IPM) strategies in different cropping systems.

## Figures and Tables

**Table 1:** The standard chemical samples in the experiment.

Compounds	CAS Number	Purity (%)	Producer
*β*-ocimene	3779-61-1	98	Toronto Research Chemicals (Vaughan, ON, Canada)
1-heptene	592-76-7	98	Bejing Ouhe Technology Co., Ltd. (Beijing, China)
aromadendrene	489-39-4	97	Sigma-Aldrich (St. Louis, MO, USA)
*trans*-2-hexen-1-al	6728-26-3	98	Bejing Ouhe Technology Co., Ltd.
*α*-farnesene	502-61-4	98	Toronto Research Chemicals
heptadecane	629-78-7	98	Bejing Ouhe Technology Co., Ltd.
*trans*-2-hexen-1-ol	928-95-0	98	Bejing Ouhe Technology Co., Ltd.
*(Z)*-3-hexen-1-ol (Leaf alcohol)	928-96-1	98	Bejing Ouhe Technology Co., Ltd.
*α*-phellandrene	99-83-2	98	Bejing Ouhe Technology Co., Ltd.
*β*-pinene	127-91-3	98	Bejing Ouhe Technology Co., Ltd.
*α*-caryophyllene	6753-98-6	98	Bejing Ouhe Technology Co., Ltd.
hexadecene	629-73-2	98	Bejing Ouhe Technology Co., Ltd.
nerolidol	7212-44-4	98	Bejing Ouhe Technology Co., Ltd.
3-methylpentanal	15877-57-3	98	Toronto Research Chemicals
n-hexane	110-54-3	97	Tianjin Fuyu Fine Chemical Co., Ltd. (Tianjin, China)
paraffin liquid	8042-47-5	99	Tianjin Yongcheng Fine Chemical Co., Ltd. (Tianjin, China)

Note: The compounds 1-heptene, aromadendrene, *trans*-2-hexen-1-ol, *α*-phellandrene, *α*-caryophyllene, and hexadecene in the table are from cotton leaves; the compounds *α*-farnesene and heptadecane are from corn leaves; and the compounds *β*-ocimene, *trans*-2-hexenal-1-al, leaf alcohol, *β*-pinene, nerolidol, and 3-methylpentanal are from both cotton leaves and corn leaves.

**Table 2:** Compositions and contents of twenty-six mixtures of plant volatiles.

Mixture	**Composition**	**Concentration (μL/mL)**
1	*β*-pinene + leaf alcohol	10 + 10
2	*β*-pinene + *trans*-2-hexen-1-al	10 + 10
3	*β*-pinene + aromadendrene	10 + 10
4	*α*-phellandrene + *trans*-2-hexen-1-ol	10 + 10
5	*α*-phellandrene + *trans*-2-hexen-1-al	10 + 10
6	*α*-phellandrene + leaf alcohol	10 + 10
7	*α*-phellandrene + *α*-farnesene	10 + 1
8	*trans*-2-hexen-1-al + leaf alcohol	10 + 10
9	*trans*-2-hexen-1-al + *trans*-2-hexen-1-ol	10 + 10
10	*trans*-2-hexen-1-al + aromadendrene	10 + 10
11	leaf alcohol + 1-heptene	10 + 10
12	leaf alcohol + *α*-farnesene	10 + 1
13	*trans*-2-hexen-1-ol + 1-heptene	10 + 10
14	*trans*-2-hexen-1-ol + nerolidol	10 + 10
15	1-heptene + nerolidol	10 + 10
16	1-heptene + *α*-farnesene	10 + 1
17	nerolidol + aromadendrene	10 + 10
18	aromadendrene + *α*-farnesene	10 + 1
19	*β*-pinene + *α*-phellandrene + *trans*-2-hexen-1-al	10 + 10 + 10
20	leaf alcohol + *trans*-2-hexen-1-ol +1-heptene	10 + 10 + 10
21	nerolidol + aromadendrene + *α*-farnesene	10 + 10 + 1
22	*β*-pinene + *trans*-2-hexen-1-al +leaf alcohol	10 + 10 + 10
23	*α*-phellandrene + *trans*-2-hexen-1-ol + 1-heptene	10 + 10 + 10
24	leaf alcohol + nerolidol + aromadendrene	10 + 10 + 10
25	*trans*-2-hexen-1-ol + nerolidol + aromadendrene	10 + 10 + 10
26	*trans*-2-hexen-1-al + 1-heptene + *trans*-2-hexen-1-ol	10 + 10 + 10

**Table 3:** Results for the chi-square tests regarding the effects of female and male of the *M. signata* adults to different combinations in the Y-tube olfactometer assays.

Combinations	Gender	*χ* ^2^	df	*p*	*p* (Bonferroni Corrected)
Mixture 1 vs. CK	Female	3.981	1	0.046	0.067
Mixture 4 vs. CK	Female	0.908	1	0.341	0.430
Mixture 12 vs. CK	Female	1.333	1	0.248	0.320
Mixture 20 vs. CK	Female	5.055	1	0.025	0.037
Mixture 22 vs. CK	Female	1.171	1	0.279	0.357
Mixture 23 vs. CK	Female	5.762	1	0.016	0.025
Mixture 25 vs. CK	Female	1.126	1	0.289	0.368
Mixture 26 vs. CK	Female	6.248	1	0.012	0.020
Mixture 4 vs. CK	Male	1.126	1	0.289	0.368
Mixture 8 vs. CK	Male	0.253	1	0.615	0.733
Mixture 19 vs. CK	Male	4.244	1	0.039	0.058
Mixture 20 vs. CK	Male	0.805	1	0.370	0.461
Mixture 22 vs. CK	Male	6.061	1	0.014	0.021
Mixture 23 vs. CK	Male	4.144	1	0.042	0.061
Mixture 24 vs. CK	Male	0.187	1	0.666	0.857
Mixture 26 vs. CK	Male	2.522	1	0.112	0.154

**Table 4:** The average number of *M. signata* adults every two days.

**Treatment**	Date						
	14 July	16 July	18 July	20 July	22 July	24 July	26 July
Mixture 1	1.7 ± 1.2 a	2.7 ± 0.3 ab	1.0 ±1.0 ab	2.3 ± 0.3 a	0.7 ± 0.7 a	1.3 ± 0.9 ab	1.0 ± 0.6 a
Mixture 19	2.0 ± 1.5 a	3.7 ± 1.3 ab	1.0 ± 0.0 ab	3.3 ± 2.3 a	0.7 ± 0.3 a	0.7 ± 0.7 b	0.3 ± 0.3 a
Mixture 20	1.7 ± 1.2 a	2.7 ± 0.9 ab	3.3 ± 1.7 a	1.3 ± 0.3 a	0.3 ± 0.3 a	2.0 ± 0.6 ab	1.3 ± 0.9 a
Mixture 22	1.7 ± 0.3 a	5.0 ± 1.2 a	1.3 ± 0.3 ab	2.0 ± 0.6 a	1.0 ± 0.6 a	1.7 ± 0.7 ab	1.0 ± 0.6 a
Mixture 23	4.0 ± 1.5 a	4.3 ± 1.2 a	2.3 ± 0.9 ab	2.3 ± 0.7 a	1.0 ± 0.6 a	3.3 ± 0.9 a	0.3 ± 0.3 a
Mixture 26	2.7 ± 0.7 a	3.3 ± 0.7 ab	3.0 ± 0.6 a	2.0 ± 1.5 a	1.3 ± 0.3 a	1.0 ± 1.0 ab	0.7 ± 0.7 a
CK	0.7 ± 0.3 a	1.0 ± 0.0 b	0.0 ± 0.0 b	0.7 ± 0.3 a	0.3 ± 0.3 a	0.7 ± 0.3 b	0.3 ± 0.3 a

Note: Data are presented as the mean ± SE. Different lowercase indicates a signifcant difference at the *p* < 0.05 level.

## Data Availability

The original contributions presented in this study are included in the article/Supplementary Materials. Further inquiries can be directed to the corresponding authors.

## Supplementary Materials

The following supporting information can be downloaded at: https://www.mdpi.com/article/10.3390/insects16121233/s1.

## Abbreviations

The following abbreviations are used in this manuscript:

EAG	Electroantennogram
SE	Standard error

## References

[1] Dudareva N., Negre F., Nagegowda D.A. (2006). Plant Volatiles: Recent Advances and Future Perspectives. Crit. Rev. Plant Sci..

[2] Pichersky E., Noel J.P., Dudareva N. (2006). Biosynthesis of plant volatiles: Nature’s diversity and ingenuity. Science.

[3] Pinto D.M., Himanen S.J., Nissinen A., Nerg A.M., Holopainen J.K. (2008). Host location behavior of *Cotesia plutellae* Kurdjumov (Hymenoptera: Braconidae) in ambient and moderately elevated ozone infield conditions. Environ. Pollut..

[4] Knudsen J.T., Gershenzon J., Dudareva N., Pichersky E. (2006). The chemistry diversity of floral scent. Biology of Floral Scent.

[5] Lou Y., Cheng J. (1997). Induced plant resistance to phytophagous insects. Acta Entomol. Sin..

[6] Bruce T.J.A., Wadhams L.J., Woodcock C.M. (2005). Insect host location: A volatile situation. Trends Plant Sci..

[7] Turlings T.C.J., Erb M. (2018). Tritrophic interactions mediated by herbivore-induced plant volatiles: Mechanisms, ecological relevance, and application potential. Annu. Rev. Entomol..

[8] Branco S., Mateus E.P., Silva M., Mendes D., Rocha S.M., Mendel Z., Schütz S., Paiva M.R. (2019). Electrophysiological and behavioural responses of the *Eucalyptus weevil*, *Gonipterus platensis*, to host plant volatiles. J. Pest Sci..

[9] Sun X.L., Li X.W., Xin Z.J., Han J.J., Ran W., Lei S. (2016). Development of synthetic volatile attractant for male *Ectropis obliqua* moths. J. Integr. Agric..

[10] Cai X.M., Bian L., Xu X.X., Luo Z.X., Li Z.Q., Chen Z.M. (2017). Field background odour should be taken into account when formulating a pest attractant based on plant volatiles. Sci. Rep..

[11] Xiu C.L., Pan H.S., Liu B., Luo Z.X., Williams L., Yang Y.H., Lu Y.H. (2019). Perception of and behavioral responses to host plant volatiles for three Adelphocoris species. J. Chem. Ecol..

[12] Yu P.Y., Wang S.Y., Yang X.K. (1996). Pyrophylloidea, Economic Insects of China, Volume 54 (II).

[13] Li J.H., Shi Y.X., Song L. (2020). The complete mitochondrial genome of an important agricultural pest *Monolepta hieroglyphica* (Coleoptera: Chrysomelidae: Galerucinae). Mitochondrial DNA Part B.

[14] Chen J., Zhang J.P., Zhang J.H., Yu F.H., Li G.W. (2007). Food preference of *Monolepta hieroglyphica*. J. Appl. Entomol..

[15] Zhang J.H., Zhang J.P., Wang P.L., Li Y. (2005). New Trends of Cotton Pests in Xinjiang and Their Control Countermeasures. China Cotton.

[16] Tian Y.H., Zhang J.P., Chen J., Ouyang D.H., Li G.W. (2007). Occurring Characteristic and Preventions and Control Strategy of *Monolepta hieroglyphica*(Motschulsky)-A New Pest of the Cotton Field in Xinjiang. Anhui Agric. Sci. Bull..

[17] LI G.W., Chen X.L. (2010). Studies on biological characteristics and population dynamics of *Monolepta hieroglyphica* in cotton in Xinjiang. China Plant Prot..

[18] Chen G.H., Yi W., Li Q., Hu H.Y. (2016). Research Progress of the *Monolepta hieroglyphica* (Motschulsky). China Plant Prot..

[19] Kogan M. (1998). Integrated pest management: Historical perspectives and contemporary developments. Annu. Rev. Entomol..

[20] Wang H.M., Bai P.H., Zhang J. (2020). Attraction of bruchid beetles *Callosobruchus chinensis* (L.) (Coleoptera: Bruchidae) to host plant volatiles. J. Integr. Agric..

[21] He W.J., Meng H.Y., Zhang Y., Zhang G., Zhi M.T., Li G.W., Chen J. (2024). Identification of candidate chemosensory genes in the antennal transcriptome of *Monolepta signata*. PLoS ONE.

[22] Cao J.Y., He W.J., Li H.Q., Zhu J.Y., Li X.G., Tian J.H., Luo M.D., Chen J. (2025). The Ultrastructure of Olfactory Sensilla Across the Antenna of *Monolepta signata* (Oliver). Insects.

[23] Gallego D., Galián J., Diez J.J., Pajares J.A. (2008). Kairomonal responses of *Tomicus destruens* (Col., Scolytidae) to host volatiles *α*-pinene and ethanol. J. Appl. Entomol..

[24] Schiestl F.P. (2010). The evolution of floral scent and insect chemical communication. Ecol. Lett..

[25] Xu Z., Zhang G., Qiu Y., Luo Z., Cai X., Li Z., Bian L., Fu N., Zhou L., Magsi F.H. (2024). Mixture of Synthetic Plant Volatiles Attracts More Stick Tea Thrips *Dendrothrips minowai* Priesner (Thysanoptera: Thripidae) and the Application as an Attractant in Tea Plantations. Plants.

[26] Bruce T.J.A., Pickett J.A. (2011). Perception of plant volatile blends by herbivorous insects-finding the right mix. Phytochemistry.

[27] Karmakar A., Mitra P., Koner A., Das S., Barik A. (2020). Fruit volatiles of creeping cucumber (*Solena amplexicaulis*) attract a generalist insect herbivore. J. Chem. Ecol..

[28] Roberts J.M., Kundun J., Rowley C., Hall D., Douglas P., Pope T.W. (2019). Electrophysiological and behavioral responses of adult vine weevil, *Otiorhynchus sulcatus* (Coleoptera: Curculionidae), to host plant odors. J. Chem. Ecol..

[29] Adhikary P., Mukherjee A., Barik A. (2015). Attraction of *Callosobruchus maculatus* (F.) (Coleoptera: Bruchidae) to four varieties of *Lathyrus sativus* L. seed volatiles. Bull. Entomol. Res..

[30] Li X., Zhang X., Xiao C. (2020). Behavioral responses of potato tuber moth (*Phthorimaea operculella*) to tobacco plant volatiles. J. Integr. Agric..

[31] Liu H., Chi D.F., Chen H.Y., Yu J., Li X.C. (2013). EAG and Behavioral Responses of *Monolepta hieroglyphica* (Motschulsky) to Several Volatile Compounds. For. Res..

[32] Cuo D.D., Zhang Z.H., Chen J., Wang S.S. (2018). Electrophysiological and behavioral responses of *Monolepta hieroglyphica* (Motschulsky) to 7 cotton and corn volatiles. Chin. Bull. Entomol..

[33] Zhang Z.H., Chen J., Tang S.Q., Zhang J., Li L. (2018). Olfactory Behavioral Response of the *Monolepta hieroglyphica* (Motschulsky) to volatiles of cotton and corn such as Dragosantol. Xinjiang Agric. Sci..

[34] Siderhurst M.S., Jang E.B. (2006). Female-biased attraction of oriental fruit fly, *Bactrocera dorsalis* (Hendel), to a blend of host fruit volatiles from *Terminalia catappa* L. J. Chem. Ecol..

[35] Tang Y., Zhou C., Chen X., Zheng H. (2013). Foraging Behavior of the Dead Leaf Butterfly, *Kallima inachus*. J. Insect Sci..

[36] Derstine N.T., Meier L., Canlas I., Murman K., Cannon S., Carrillo D., Cooperband M.F. (2020). Plant Volatiles Help Mediate Host Plant Selection and Attraction of the *Spotted Lanternfly* (Hemiptera: Fulgoridae): A Generalist with a Preferred Host. Environ. Entomol..

[37] Yang G., Zhang Y.N., Gurr G.M. (2016). Electroantennogram and behavioral responses of *Cotesia plutellae* to plant volatiles. Insect Sci..

[38] Cha D.H., Linn C.E., Teal P.E.A., Aijun Z., Roelofs W.L., Loeb G.M. (2011). Eavesdropping on plant volatiles by a specialist moth: Significance of ratio and concentration. PLoS ONE.

[39] Du J.W. (2001). Plant-insect Chemical Communication and Its Behavior Control. Physiol. Mol. Biol. Plants.

[40] Zhao D.X., Lu F.P., Mo S.S., Wang A.P. (2006). The physiological activities of several plant volatiles on the antennae potential of adult *Brontispa longissima* (Gestro). Chinesa J. Trop. Crops.

[41] Ning T., Fan J.T., Fang Y.L., Sun J.H. (2006). Changes in contents of host volatile terpenes under different damaged states and electroantennogram response of *Monochamus alternatus* Hope to these volatiles. Acta Entomol. Sin..

[42] Dierks A., Fischer K. (2008). Feeding responses and food preferences in the tropical, fruit-feeding butterfly, *Bicyclus anynana*. J. Insect Physiol..

[43] Yang Y.T., Su Q., Shi L.L., Chen G., Zeng Y., Shi C.H., Zhang Y.J. (2019). Electrophysiological and behavioral responses of *Bradysia odoriphaga* (Diptera: Sciaridae) to volatiles from its Host Plant, Chinese Chives (Allium tuberosum Rottler ex Spreng). J. Econ. Entomol..

[44] Zhang Q.H., Schlyter F., Chen G.F., Wang Y.J. (2007). Electrophysiological and Behavioral Responses of Ips subelongatus to Semiochemicals from Its Hosts, Non-hosts, and Conspecifics in China. J. Chem. Ecol..

[45] Tang R., Zhang P.J., Zhang N.Z. (2018). Electrophysiological and behavioral responses of male fall webworm moths (*Hyphantria cunea*) to Herbivory-induced mulberry (*Morus alba*) leaf volatiles. PLoS ONE.

[46] Thöming G., Knudsen G.K. (2014). Attraction of pea moth *Cydia nigricana* to pea flower volatiles. Phytochemistry.

[47] Shrivastava G., Rogers M., Wszelaki A., Panthee D.R., Chen F. (2010). Plant volatiles-based insect pest management in organic farming. Crit. Rev. Plant Sci..

[48] Romero P., Ibarra-Juárez L.A., Carrillo D., Guerrero-Analco J.A., Kendra P.E., Kiel-Martínez A.L., Guillén L. (2022). Electroantennographic Responses of Wild and Laboratory-Reared Females of *Xyleborus affinis* Eichhoff and *Xyleborus ferrugineus* (Fabricius) (Coleoptera: Curculionidae: Scolytinae) to Ethanol and Bark Volatiles of Three Host-Plant Species. Insects.

[49] Li W.Z., Yang L., Shen X.W., Yuan Y.H., Yuan G.H., Luo M.H., Guo X.R. (2013). Prescription screening and field evaluation of broad spectrum attractants of scarab beetles from Ricinus communis. Chin. J. Eco-Agric..

[50] Li X., Ju Q., Jin Q., Jiang X.J., Su W.H., Zhang G.L., Xie M.H., Qu M.J. (2015). Field Trapping Efficacy of Different Lures and Traps on *Holotrichia parallela* (Coleoptera, Scarabaeidae, Melolonthinae). J. Peanut Sci..

[51] Honda K., Ômura H., Hayashi N. (1998). Identification of floral volatiles from Ligustrum japonicum that stimulate flower-visiting by cabbage butterfly, *Pieris rapae*. J. Chem. Ecol..

[52] Ômura H., Honda K. (2009). Behavioral and electroantennographic responsiveness of adult butterflies of six nymphalid species to food-derived volatiles. Chemoecology.

[53] Knudsen G.K., Bengtsson M., Kobro S., Jaastad G., Witzgall P. (2008). Discrepancy in laboratory and field attraction of apple fruit moth *Argyresthia conjugella* to host plant volatiles. Physiol. Entomol..

[54] Knudsen G.K., Tasin M. (2015). Spotting the invaders: A monitoring system based on plant volatiles to forecast apple fruit moth attacks in apple orchards. Basic Appl. Ecol..

[55] Wei J., Kang L. (2011). Roles of *(Z)*-3-hexenol in plant-insect interactions. Plant Signal. Behav..

[56] Reinecke A., Ruther J., Tolasch T. (2002). Alcoholism in cockchafers: Orientation of male *Melolontha melolontha* towards green leaf alcohols. Die Naturwissenschaften.

[57] Ruther J. (2004). Male-biassed response of garden chafer, *Phyllopertha horticola* L., to leaf alcohol and attraction of both sexes to floral plant volatiles. Chemoecology.

[58] Reddy G.V.P., Guerrero A. (2004). Interactions of insect pheromones and plant semiochemicals. Trends Plant Sci..

[59] Li J.G., Jin Y.J., Luo Y.Q., Xu Z.C., Chen H.J. (2003). Leaf Volatiles from Host Tree Acer negundo: Diurnal Rhythm and Behavior Responses of *Anoplophora glabripennis* to Volatiles in Field. Axiaa Bot. Sin..

[60] Chen Y.S., Li F.X., Wen D.H. (2014). Review of Research on Attractant from Plants to *Monochamus alternatus* Hope. J. Henan Agric. Sci..

[61] Zhang Y., Lu Q. (2016). Research on the EAG and behavial responses of *Asias halodendri* to the plant Prunus armeniaca’ s volatiles. J. Environ. Entomol..

[62] Ze S.X., Zhao N., Wang D.W., Ji M., Yang B. (2013). The efficacy of *Trans*-2-hexenal as an Attractant for adult *Monochamus alternatus*. For. Pest Dis..

[63] Miller D.R., Borden J.H. (1990). *β*-Phellandrene: Kairomone for pine engraver, *Ips pini*(Say) (Coleoptera: Scolytidae). J. Chem. Ecol..

[64] Zhang Y., Lu Q., Zhu G.P., LIi M. (2015). Research on the EAG and behavioral responses of *Chlorophorus diadema* to eleven Vitis vinifera and Prunus armeniaca volatiles. Chin. J. Appl. Entomol..

[65] Qiu H., Zhao D., Fox E.G.P., Ling S., Qin C., Xu J. (2022). Chemical Cues Used by the Weevil Curculio chinensis in Attacking the Host Oil Plant Camellia oleifera. Diversity.

[66] Li S., Gao W., Chen X.C., Zhou Y.T., Cui W.C. (2016). Electroantennogram response of *Anoplophora glabripennis* (Motsch.) to Acer negundo volatiles. For. Pest Dis..

