# Supplementary material for: Plant Volatile Organic Compounds Attractive to Monolepta signata (Olivier)
Source: Insects. 2025 Dec 6;16(12):1233. doi: 10.3390/insects16121233 (PMC12733953; doi:10.3390/insects16121233)
Supplement: Supplementary file 1 [file insects-16-01233-s001.zip › insects-3911897-supplementary/Supplementary File(s)/Figure S1.pdf]

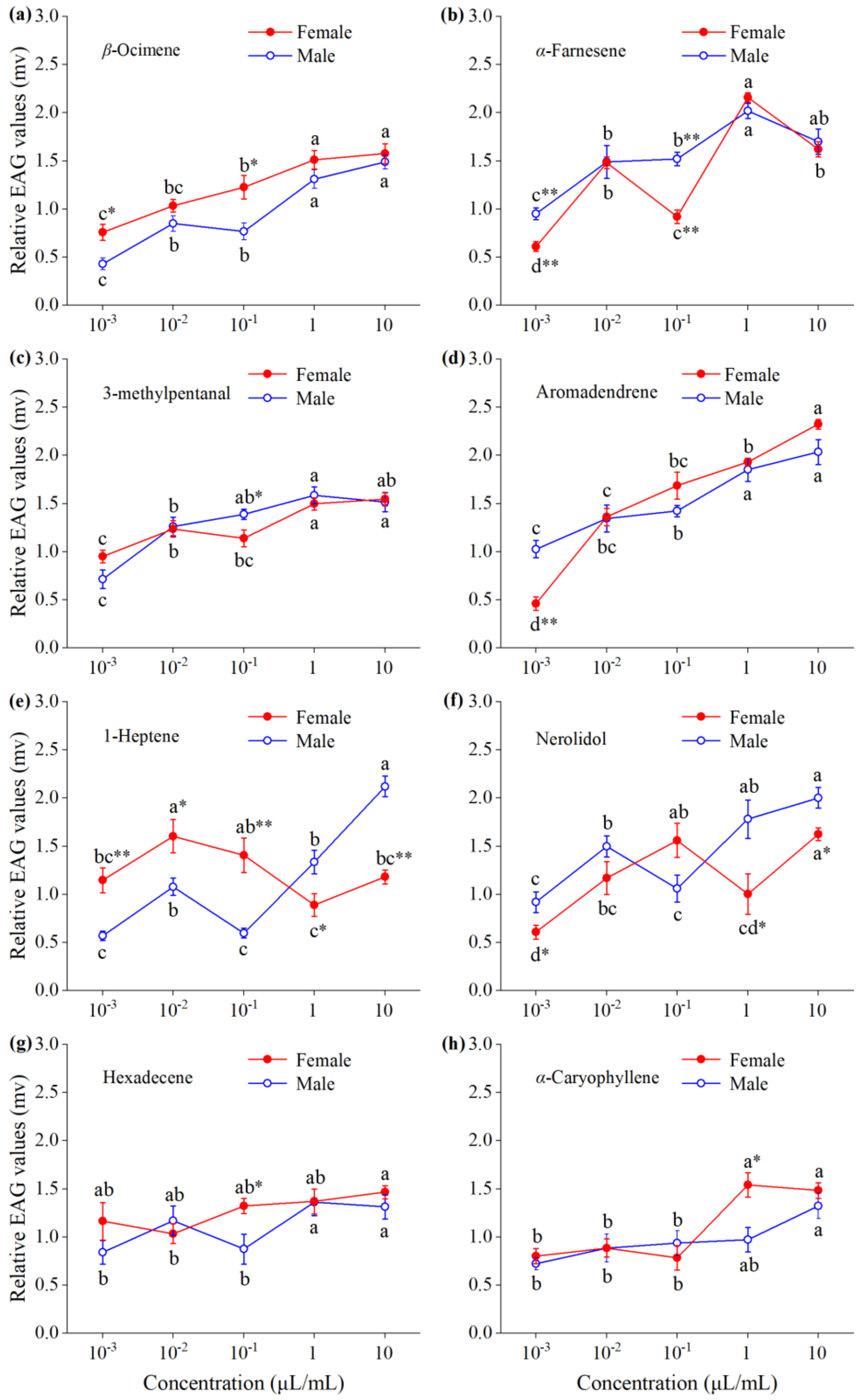
(a) β-Ocimene
(b) α-Farnesene
(c) 3-methylpentanal
(d) Aromadendrene
(e) 1-Heptene
(f) Nerolidol
(g) Hexadecene
(h) α-Caryophyllene
Female
Male
Relative EAG values (mv)
Concentration (μL/mL)
10-3
10-2
10-1
1
10

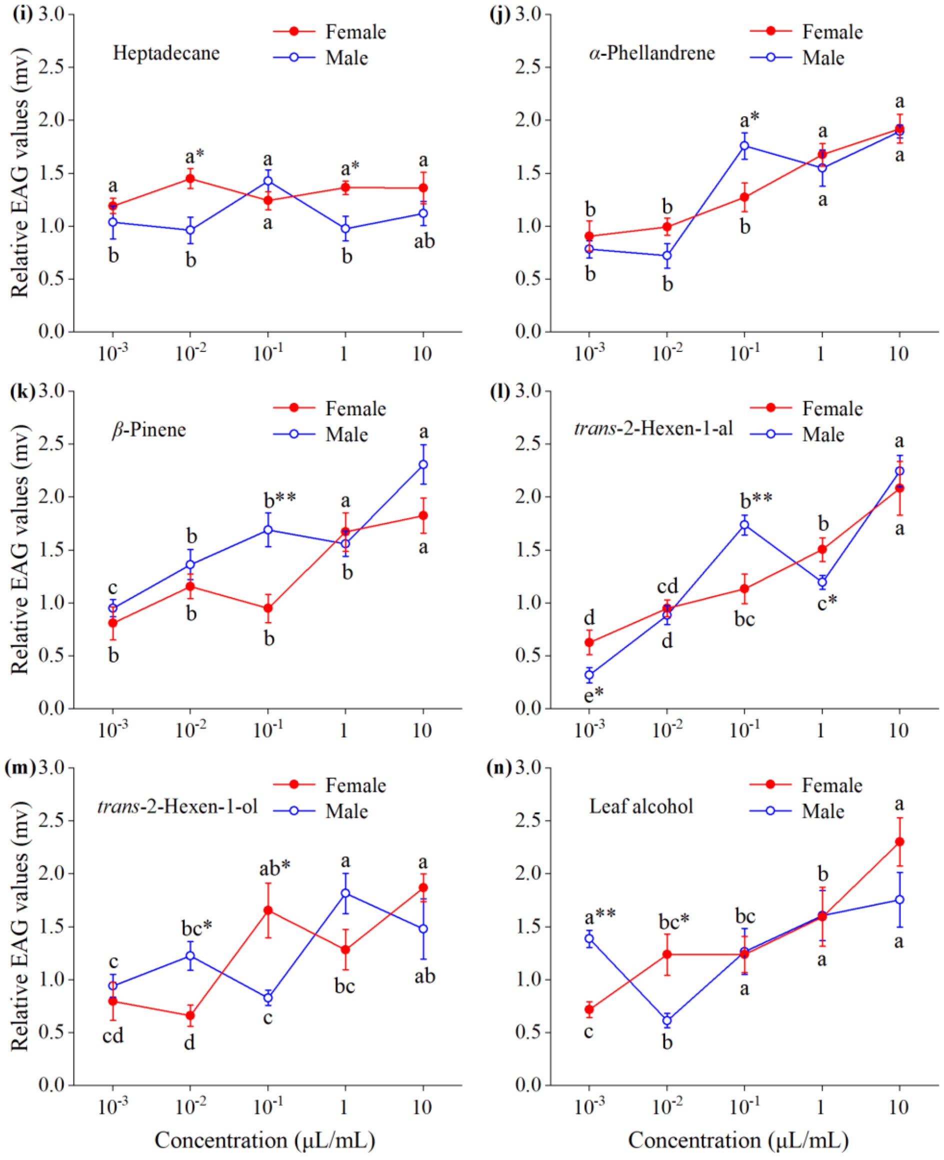

Figure S1. Relative EAG response of *Monolepta signata* of 14 kind of compounds. Small letters of male and female worms Duncan multiple comparison EAG response values of different concentrations for same compounds，* indicates that the female and male antennae on the same volatile EAG reaction significant difference t test in $P < 0.05$ levels ． ** indicates that significant different t test in $P < 0.01$ level.
