# Supplementary material for: Plant Volatile Organic Compounds Attractive to Monolepta signata (Olivier)
Source: Insects. 2025 Dec 6;16(12):1233. doi: 10.3390/insects16121233 (PMC12733953; doi:10.3390/insects16121233)
Supplement: Supplementary file 1 [file insects-16-01233-s001.zip › insects-3911897-supplementary/Supplementary File(s)/Figure S2.pdf]

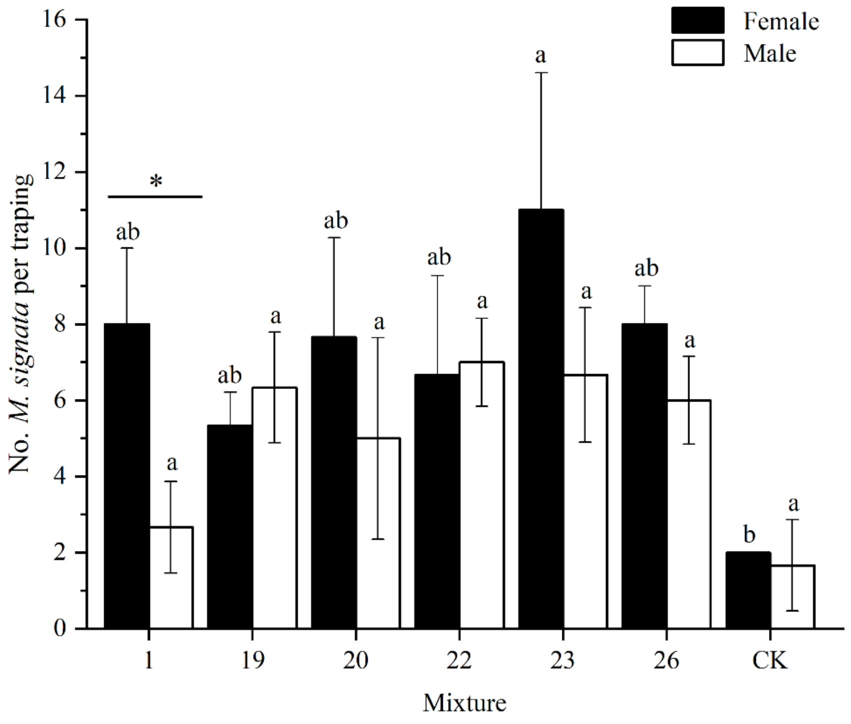

Figure S2. Field trapping effects of six plant volatile mixtures on female and male *M. signata* adults. Different lowercase letters above the bars of the same sex indicate significant differences among attractant mixtures (Duncan's multiple-range test, $P < 0.05$). An asterisk (*) indicates a significant **difference** between female and male catches for the same attractant (*t*-test, $P < 0.05$).
