# Supplementary material for: Plant Volatile Organic Compounds Attractive to Monolepta signata (Olivier)
Source: Insects. 2025 Dec 6;16(12):1233. doi: 10.3390/insects16121233 (PMC12733953; doi:10.3390/insects16121233)
Supplement: Supplementary file 1 [file insects-16-01233-s001.zip › insects-3911897-supplementary/Supplementary File(s)/Figure S3.pdf]

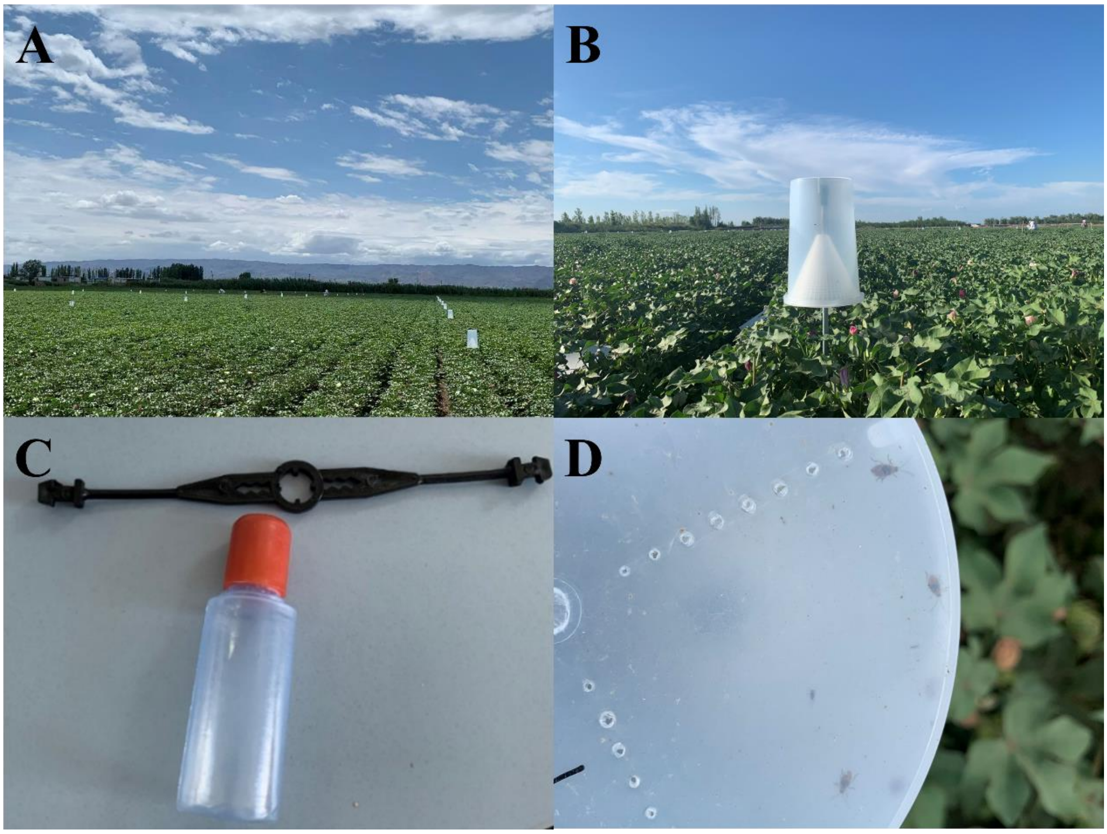

Figure S3. Field trapping setup for *M. signata* adults. (A) Layout of traps, evenly distributed across the crop plots. (B) Trap placement height above the plant canopy. (C) Polyethylene slow-release vial containing the plant volatile mixture used as the lure. (D) *M. signata* adults captured in a trap.
