# Supplementary material for: Plant Volatile Organic Compounds Attractive to Monolepta signata (Olivier)
Source: Insects. 2025 Dec 6;16(12):1233. doi: 10.3390/insects16121233 (PMC12733953; doi:10.3390/insects16121233)
Supplement: Supplementary file 1 [file insects-16-01233-s001.zip › insects-3911897-supplementary/Supplementary File(s)/Figure S4.pdf]

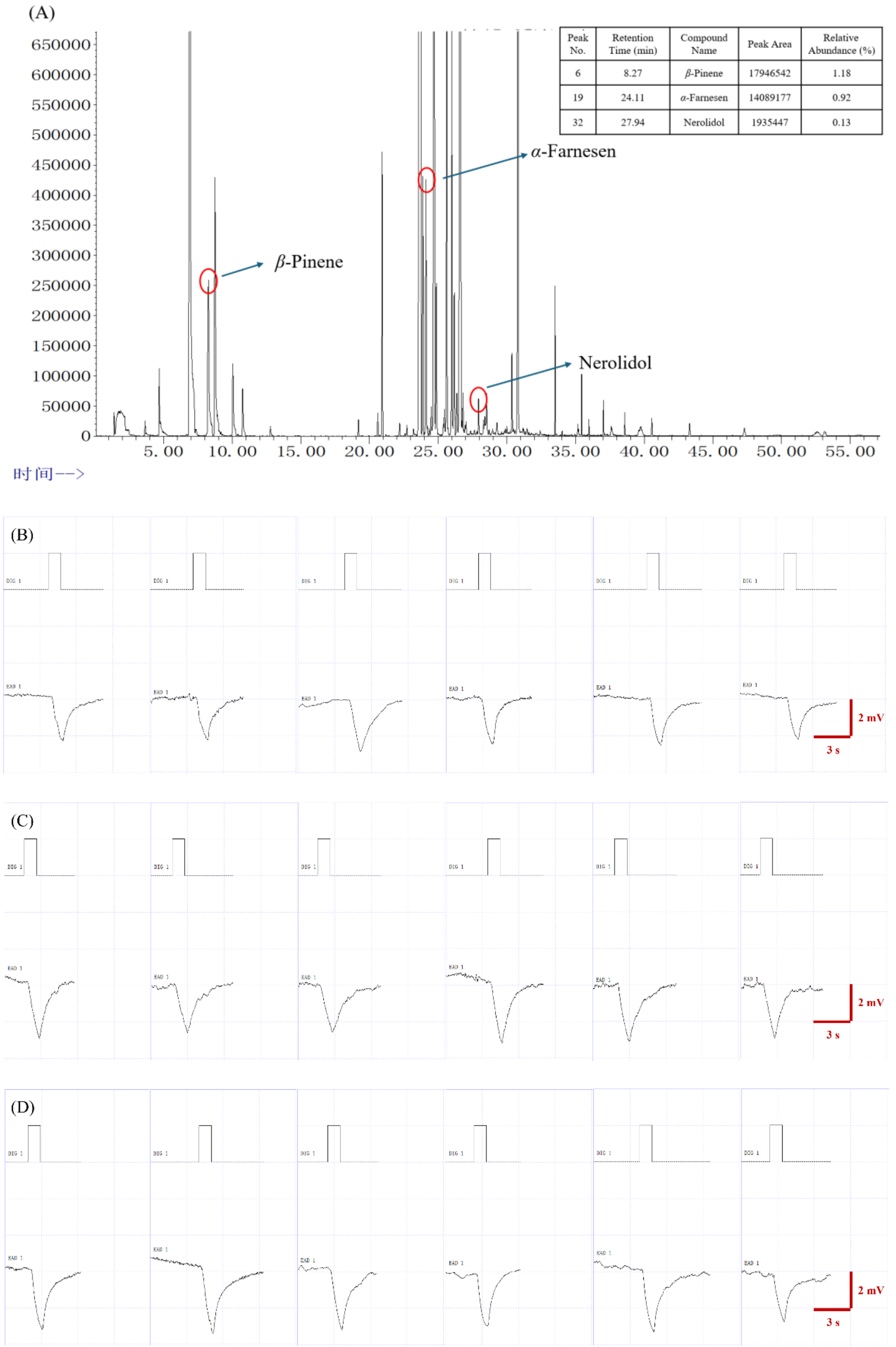

Peak No.	Retention Time (min)	Compound Name	Peak Area	Relative Abundance (%)
6	8.27	*β*-Pinene	17946542	1.18
19	24.11	*α*-Farnesen	14089177	0.92
32	27.94	Nerolidol	1935447	0.13

Figure S4. GC–MS chromatographic identification of volatile compounds emitted from cotton plants(A). The table lists the retention time, compound identity, peak area, and relative peak area (%) of each component detected. Compounds were identified by matching mass spectra with the NIST library

and confirmed by retention indices when available. **Representative EAG recordings of female *M. signata* antennae responding to Mixture 1 (B), Mixture 22 (C) and Mixture 25.** Each panel shows six independent biological replicates recorded from different antennae.
